# Recent trends in US government healthcare & behavioral health workforce departures

**DOI:** 10.1093/haschl/qxag032

**Published:** 2026-02-07

**Authors:** Nichole Fusilier, Elisabeth Stelson, Janette Dill

**Affiliations:** School of Public Health, University of Minnesota, 420 Delaware St SE, Minneapolis, MN 55455, United States; School of Public Health, Washington University, St. Louis, MO 63130, United States; School of Public Health, University of Minnesota, 420 Delaware St SE, Minneapolis, MN 55455, United States

**Keywords:** government workers, federal workforce, state workforce, local workforce, public health, healthcare workforce, behavioral health workforce

## Abstract

**Introduction:**

Healthcare and behavioral health professionals employed by local, state, and federal governments are essential to maintaining public health infrastructure, ensuring access to care, and responding to emergencies. Despite their importance, limited research has examined how recent policy, budgetary, and labor market changes are influencing their employment stability and retention within government sectors.

**Methods:**

This study used longitudinal data from the Current Population Survey (2015-2025) to examine employment transitions among government-employed healthcare and behavioral health workers. We estimated the predicted probabilities of (1) transitions from government to non-governmental employment and (2) full exits from the labor force.

**Results:**

Historically, federal healthcare and behavioral health workers had the lowest exit rates from government employment, but their probability of leaving government employment rose sharply in 2024-2025, converging with state and local levels (8%). Healthcare workers were consistently more likely than behavioral health workers to transition out of government roles, though both groups experienced higher exit rates in 2024-2025. Federal employees also exhibited a modest increase in labor force exits, from 2.5%-3% to 3.8% in 2024-2025.

**Conclusion:**

These trends suggest increasing instability in the government-employed health workforce.

Key Takeaways:

- Exits from government employment among healthcare and behavioral health workers increased sharply in 2024/2025.
- Federal workers, who historically had the lowest exit rates, experienced a notable rise in departures.
- Healthcare workers consistently had higher exit rates than behavioral health workers.

## Introduction

Healthcare and behavioral health professionals employed by local, state, and federal governments are integral to the delivery of health services in the United States, spanning community-based prevention programs to large integrated systems of care.^1,2^ Although the effects of federal funding shifts and workforce downsizing have been well documented,^3-5^ far less is known about how these changes have shaped employment among government-employed healthcare and behavioral health workers.^6-8^ This study addresses that gap by analyzing employment patterns in these workforces over the past decade, with particular attention to emerging shifts in 2025.

### Government-employed healthcare and behavioral health workforce

Although the U.S. healthcare system is largely privatized, thousands of health professionals are employed directly as government workers, many providing critical services that are often overlooked.^9^ For example, over 310 000 workers in the federal workforce are in healthcare occupations.^10^ Many additional thousands of healthcare and behavioral health workers are employed by state and local governments. These workers include public health nurses, health educators, and behavioral health specialists who provide care in public hospitals, county health departments, schools, and state-run mental health facilities. Examples of large county-run safety net hospitals that employ thousands of healthcare and behavioral health workers include Los Angeles County + USC Medical Center, one of the largest public hospitals in the country, operated by the Los Angeles County Department of Health Services,^11^ Cook County Health, which includes John H. Stroger Jr. Hospital and Provident Hospital, both operated by Cook County to serve residents regardless of ability to pay,^12^ and Hennepin County Medical Center in Minnesota.^13^ Philadelphia Department of Public Health operates eight primary care clinics across the city.^14^ Examples of state-run hospitals include University Hospital, which is operated by the state of New Jersey and serves as the principal teaching hospital for Rutgers New Jersey Medical School, and Central State Hospital, a state-operated psychiatric facility run by the Virginia Department of Behavioral Health and Developmental Services. State agencies also often deploy social workers and mental health counselors in community- and school-based programs, such as South Carolina's initiative that recently doubled the number of counselors placed in schools.^15^

At the federal level, important government-operated systems like the Veterans Health Administration (VA) and the Indian Health Service (IHS) employ tens of thousands of healthcare and behavioral health professionals. The VA, the nation's largest integrated health care system, serves more than 8 million veterans annually.^16^ Similarly, IHS operates facilities across 35 states to serve over 2 million American Indian and Alaska Native people, employing clinicians such as nurses, psychiatrists, social workers, and substance use disorder counselors to address both physical and behavioral health needs.^17^

Recent federal budget reductions and administrative restructuring, however, have disrupted these workforces. At the federal level, the Department of Health and Human Services (HHS) is undergoing a large-scale reorganization that is expected to reduce its workforce by nearly 25%, decreasing from approximately 82 000 to 62 000 full-time employees.^18^ Key agencies such as the CDC, FDA, NIH, and SAMHSA are facing significant staff reductions,^19^ while many federally funded grant programs that support community-based behavioral health services and addiction treatment are being eliminated or scaled back.^8^ These reductions are reverberating across states and municipalities. In Dallas County, Texas, for example, federal grant cuts prompted layoffs of more than 20 employees and the cancellation of dozens of immunization clinics that had been designed to reach underserved populations.^20^

Federal health systems, including the VA and IHS, have also experienced funding cuts and staff layoffs. Between January and June 2025, the VA lost nearly 17 000 employees.^21^ Although an earlier plan called for eliminating 80 000 positions, current reductions are being implemented through attrition, early retirements, and hiring freezes, with a revised goal of 30 000.^22^ Given staffing shortages already reported within the VA,^23^ further staff eliminations could compromise quality of care and exacerbate long wait times. Similarly, the IHS continues to face chronic vacancy rates of around 30%,^24^ with proposed layoffs generating strong opposition from tribal leaders concerned about diminished access to essential services.^25^

Despite media attention to selected programmatic or agency-level impacts, less is known about how these policy changes are influencing overall workforce trends across government healthcare and behavioral health sectors. To address this gap, we analyzed data from the longitudinal panel of the Current Population Survey (IPUMS CPS) to assess exits from government employment among healthcare and behavioral health workers between 2015 and 2025. Using ten-month cohorts (October–July), we examined two outcomes: (1) exits from government employment with continued labor force participation, indicating transitions to non-governmental roles, and (2) exits from the labor force entirely.

## Data and methods

### Sample

This study uses data from the Current Population Survey (CPS), a monthly household survey conducted jointly by the U.S. Census Bureau and the U.S. Bureau of Labor Statistics. We use IPUMS CPS, which harmonizes microdata from the CPS.^26^ In addition to demographics, this survey collects information related to employment status, workforce industry, and occupation type, which are central points of analysis for this study. The CPS collects data from households for a total of 8 months, where respondents are interviewed for four consecutive months, then not interviewed for the following eight months, and finally interviewed again for the next four consecutive months; in total, the panel is 16 months long.

The sample included adults over the age of eighteen who were employed as (1) a local, state, or federal government worker at any period during the survey panel, and (2) worked in a healthcare or behavioral health occupation during any point of the survey period. We constructed ten-month observation cohorts spanning October through July for each year from 2015 to 2025. Each cohort was designed to begin in October, the month preceding a presidential election, in order to measure job transitions occurring in November following the election. Cohorts ended in July to align with the most recent month of data available in IPUMS CPS at the time of analysis (July 2025). Our sample included 8138 individuals who worked in a behavioral health occupation, 17 143 individuals who worked in a healthcare occupation, and 404 individuals who worked in both a behavioral health and healthcare occupation during the survey panel. The total number of observations for all individuals in the sample was 66 187.

### Measures

We had two primary outcome variables. First, we measured whether an individual *transitioned from government employment to non-government employment*. Exits from government employment were identified when an individual reported working in a government setting during the previous month but not in the current month. The second outcome variable was whether an individual employed as a government healthcare or behavioral health worker *exited the labor market*, either because they were unemployed or had chosen not to be employed.

Our key independent variables were *whether someone was employed in local, state, or federal government*, and whether they were a *healthcare worker* or a *behavioral health worker*. The occupations that were identified as healthcare and behavioral health occupations were identified by US Census codes and are included in Appendix Table S1. Additional control variables included were *race and ethnicity*, which were categorized as: White (reference category), Black, Latino, Asian, and Other/Multiracial. We also controlled for whether an individual was a *U.S. citizen.* Other demographic variables included whether an individual was *female*, *married*, *had children*, and whether they *lived in a metropolitan area*. We included six *educational categories*: no college, some college, associate degree, bachelor's degree, master's degree, or professional or doctoral degree. *Age* was grouped into three categories: 18-35 (reference category), 36-50, and 51 and older. These demographic variables have been shown to be related to work transitions in past research.^27,28^ We also included state fixed effects in our models to control for time-invariant differences across state employment contexts that might impact government employment and employment transitions.

#### Analyses

**Figure 1.**
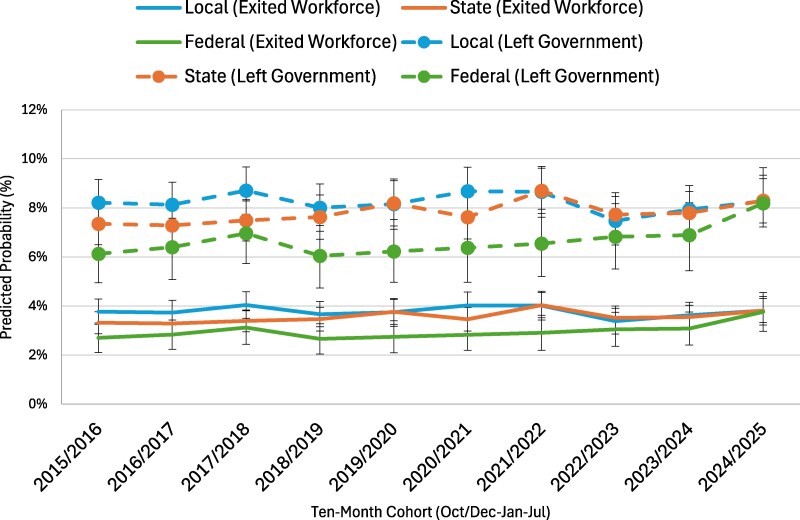
Predicted probability of exit from local, state, and federal government employment. *Source:* Current Population Survey (IPUMS CPS), 2015–2025. *Note*: Predicted probabilities were estimated based on logit models that control for sociodemographic factors (full models are included in Appendix Table S2).

**Figure 2.**
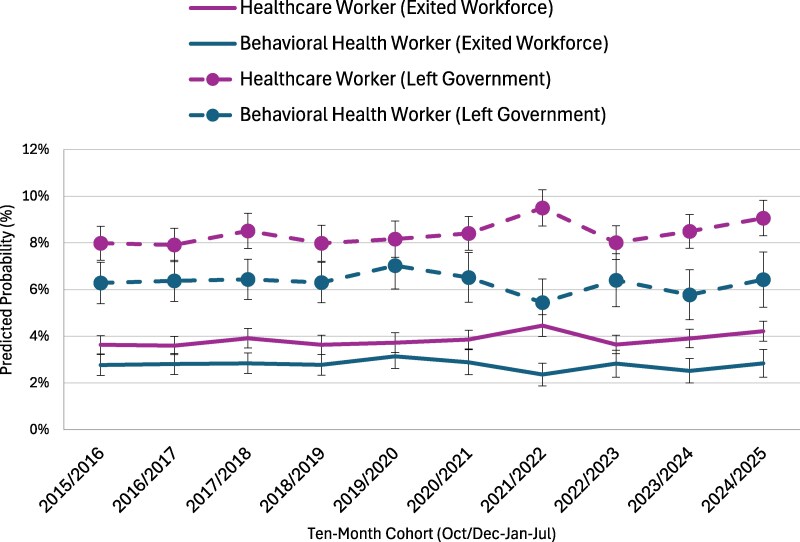
Predicted probability of exit from governmental employment by occupation. *Source:* Current Population Survey (IPUMS CPS), 2015–2025. *Note*: Predicted probabilities were estimated based on logit models that control for sociodemographic factors (full models are included in Appendix Table S2).

We estimated logistic regression models to examine two distinct types of workforce transitions among governmental employees: (1) leaving government employment for a job in another sector and (2) exiting the labor market entirely. Each model controlled for a comprehensive set of demographic and contextual characteristics, including race and ethnicity, citizenship status, gender, educational attainment, and urban or rural residence, among other factors. Following model estimation, we used the *margins* post-estimation command in Stata 18 to calculate adjusted predicted probabilities for each outcome, holding covariates at their observed values. Figure 1 presents the predicted probabilities of leaving government employment or the workforce among local, state, and federal workers, while Figure 2 displays comparable estimates for healthcare and behavioral health workers employed in government settings. Together, these figures illustrate patterns of workforce retention and exits across both institutional and occupational contexts. The complete regression results, including coefficients and standard errors for all predictors, are provided in Appendix Table S2. Additional analyses, including examining differences across workers with different levels of education and non-government workers, are included in the Appendix. All analyses were weighted using WTFINL and were conducted using Stata 18.

## Results

### Description of sample

Table 1 presents descriptive statistics for government-employed behavioral health workers, healthcare workers, and those employed in both sectors. Most workers in the sample were employed by the state government, both among those working in behavioral health and healthcare (46% and 44%, respectively), whereas federal employment was more common among healthcare workers (22%) than among behavioral health workers (13%). Across groups, the majority of workers were White (approximately 60%), followed by Black (16%-22%) and Latino (11%-12%) workers, with smaller shares identifying as Asian or American Indian/Alaska Native, Pacific Islander, other, or multiracial. Women comprised more than three-quarters of each workforce, and most workers lived in metropolitan areas. Educational attainment differed across groups: behavioral health workers are more highly educated overall, with roughly 70% holding at least a bachelor's degree—most commonly a master's degree—whereas healthcare workers have a larger share with less than a bachelor's degree and a more even spread across education levels. The average age was mid-40 s across groups, with roughly one-third under age 35, one-third between 36 and 50, and one-third over 50. Marriage and parenthood were common across all groups, with about half of workers married and nearly half reporting children.

**Table 1.** Description of sample.

	Behavioral health gov’t workers		Healthcare gov’t workers		Behavioral health & healthcare gov’t workers^a^	
	*n* = 8138		*n* = 17 143		*n* = 404	
	#	%	#	%	#	%
Worker Class						
Local	3337	41.0	5847	34.1	134	33.2
State	3742	46.0	7457	43.5	194	48.0
Federal	1059	13.0	3839	22.4	76	18.8
Race/Ethnicity & Citizenship						
White	4949	60.8	10 695	62.4	239	59.2
Black	1541	18.9	2699	15.7	84	20.8
Latino	989	12.2	1912	11.2	49	12.1
Asian	281	3.5	1231	7.2	15	3.7
AI/PI/AN/Other/Multi	378	4.6	606	3.5	17	4.2
U.S. Citizen	7959	97.8	16 481	96.1	400	99.0
Education						
No College	689	8.5	3071	17.9	37	9.2
Some College	731	9.0	2458	14.3	42	10.4
Associate Degree	583	7.2	2801	16.3	38	9.4
Bachelor's Degree	2661	32.7	4493	26.2	107	26.5
Master's Degree	3042	37.4	2621	15.3	143	35.4
Professional or Doctoral Degree	432	5.3	1699	9.9	37	9.2
Other Demographic Characteristics						
Married	4382	53.8	9597	56.0	212	52.5
Has Children	3777	46.4	8253	48.1	187	46.3
Female	6387	78.5	13 124	76.6	313	77.5
Live in Metropolitan Area	6507	80.0	13 599	79.3	309	76.5
Age						
18-35	2569	31.6	5425	31.6	111	27.5
36-50	2967	36.5	5669	33.1	152	37.6
51+	2602	32.0	6049	35.3	141	34.9

*Source:* Current Population Survey (IPUMS CPS), 2015-2025.

*Note:* Table includes the first observation for each worker in a behavioral health or healthcare occupation within a governmental setting during the survey panel.

^a^Some individuals changed occupations between observation months during the survey panel, where they were in a behavioral health occupation in one month and a healthcare occupation in a different month. We elected to report these individuals separately so that they were not overcounted in either occupation group.

### Labor market transitions among local, state, and federal healthcare and behavioral health workers

Figure 1 displays the predicted probabilities of workforce exits and transitions among governmental healthcare and behavioral health workers between 2015/2016 and 2024/2025, disaggregated by government level (local, state, and federal). Two types of transitions are shown: leaving government employment (dashed lines) and exiting the workforce entirely (solid lines). Across the period, local government workers consistently exhibited the highest likelihood of leaving government employment, with probabilities fluctuating between approximately 8% and 9%, followed closely by state workers, whose rates ranged from about 7% to 8.7%. Federal government workers had notably lower probabilities of leaving government, generally between 6% and 7%, until a sharp increase around 2024/2025 brought their rate closer to 8%, matching state and local levels.

Patterns of exiting the workforce (due to unemployment or exiting the labor force) were lower overall but showed similar relative differences by government level. Healthcare and behavioral health workers employed in local government maintained probabilities around 3.5%-4%, while state workers followed a nearly identical trajectory, hovering near 3%-4%. Federal workers consistently had the lowest rates of labor force exit, ranging between 2.5% and 3%, until a modest uptick in 2024/2025. Taken together, the figure suggests that while local and state employees are generally more likely to transition out of government or the workforce altogether, federal employees experienced a late but notable convergence toward higher exit probabilities in the most recent years of observation.

### Labor market transitions by occupational group

Figure 2 illustrates the predicted probabilities of workforce exits and transitions among governmental workers by occupational groups—healthcare and behavioral health—between 2015/2016 and 2024/2025. Two categories of transitions are displayed for each group: leaving government employment (dashed lines) and exiting the workforce entirely (solid lines). Across the full period, healthcare workers consistently showed higher probabilities of leaving government employment than behavioral health workers. The probability that healthcare workers left government ranged between roughly 8% and 9%, with a small dip around 2018/2019 and a noticeable peak approaching 10% in 2021/2022, followed by a slight decline and renewed increase by 2024/2025. In contrast, behavioral health workers exhibited a more stable pattern, with probabilities of leaving government ranging between 6% and 6.5% and a modest dip during 2021/2022.

When examining exits from the workforce entirely, the probabilities were lower overall and followed a similar relative ranking by occupation. Healthcare workers had workforce exit rates fluctuating between approximately 3.5% and 4.5%, while behavioral health workers consistently had the lowest rates, generally between 2.5% and 3%. Both groups saw a small increase in workforce exits in 2024/2025, following relatively stable trends earlier in the decade. Overall, the figure highlights that healthcare workers employed in government settings have been consistently more likely than behavioral health workers to leave government positions or exit the workforce altogether, with both groups experiencing a modest uptick in turnover probabilities in the most recent period.

## Discussion

Leveraging longitudinal panel data from the Current Population Survey, we analyzed government healthcare and behavioral health workforce trends between 2015 and 2025. Specifically, we assessed trends in transitions from government employment to non-governmental roles as well as leaving the labor market entirely over this 10-year period. Our findings indicate growing volatility in both healthcare and behavioral health workforces across all levels of government, starting during the COVID-19 pandemic. While exits lessened for both workforces and across government levels in the first years since the start of the pandemic, a sharp uptick in both transitions out of government and labor market exits is already visible with the available 2025 data. This trend is strongest among those employed at the federal level and particularly federal healthcare workers, such as those who may be employed at the VA or with the IHS. This trend may signal deepening instability in what was once considered a more secure and stable segment of the public sector workforce.

Healthcare and behavioral health services are largely privatized in the United States, and likely for this reason, literature on these workforces employed by the government is thin.^29^ While the exact size of the government healthcare and behavioral health workforce across federal, state, and local settings is unknown, it is considerable and provides essential services to veterans, indigenous communities, and people needing to access the limited safety net services provided within the federalist US healthcare system.^29^ Over 310 000 federal workers are employed in healthcare occupations, and many more work in state-run facilities and county health clinics, among other settings.^10,11^

As such, the trends in workforce reductions we identify in this study carry significant implications for service delivery and public health capacity. The U.S. Department of Health and Human Services projects that within five years, the U.S. will have fewer than 65% of the addiction treatment counselors it needs, and rural communities are expected to face registered nurse shortfalls exceeding 20%.^30^ Primary care physicians will fall short by the 26% required to keep up with projected increased demands.^30^ These projections were estimated even before 2025, and thus are likely be even greater when considered in the context of this study. While these projected workforce shortfalls pertain to both the public and private sector, persistent reductions in the government healthcare and behavioral health workforce risk further constraining service availability, heightening worker burnout, and diminishing care quality. Organizational studies indicate that even when patient-provider staffing ratios are regulated and maintained, staff attrition increases job demands for remaining staff, which consequently increases additional staff attrition, thereby creating a negative feedback loop that can reduce quality of care and access to care.^31-33^

Evidenced in both the analyses presented here and elsewhere, the United States experienced a decrease in healthcare in 2020 driven by COVID-19 exposure risks and recovery, psychological responses to stress and moral injury, diminished working conditions with insufficient organizational supports, and competing personal needs during the pandemic.^28,34,35^ While the United States has built back these workforces amidst a population with rising care needs associated with an aging population and increasing behavioral health challenges,^36^ it is imperative that we seek to expand government healthcare and behavioral health job opportunities. At a time when the country needs to be reinvesting in and rebuilding these workforces, new federal policies and practices, which dramatically shape state and local agency resources, appear to be driving higher numbers of these workers to leave government work or the workforce altogether.

### Strengths and limitations

This study is the first to evaluate employment trends in the government healthcare and behavioral health workforce that extends to 2025, a time in which substantial changes in federal funding, social policies, and layoffs are defining the healthcare and behavioral health landscape in the United States. With this population-based survey, which follows participants over the course of 16 months and contains detailed occupational and industry codes, we are able to identify a sample of government healthcare and behavioral health workers and examine their employment over time. The rapid availability of data additionally allows us to observe very recent trends.

That said, this study has several limitations that are important to consider. First, occupation and industry codes in the CPS lack precision. While we believe we identified the vast majority of healthcare and behavioral health government workers, measurement error is still possible, and for this reason, industry and occupation codes used in this analysis have been made available in Appendix A. Second, nonresponse bias may have also shaped our results, and individuals who opt to participate when randomly selected may be different from those who decline participation. However, given the reliance on scientific research for workers in this analytic sample by virtue of their jobs, nonresponse bias may be less of a concern than for the study as a whole, which has experienced declining response rates over the past decade.^37^ Third, we were only able to observe trends until July 2025, the last possible date for which data was made publicly available. Future studies should continue to monitor the observed trends in workforce instability that are already apparent at the beginning of 2025. Last, it is important to note that the CPS does not provide data on the reasons participants choose to transition their jobs, leave the workforce, or remain in their jobs. These reasons likely vary over time, responding to large social, political, and economic forces, such as the COVID-19 pandemic and reduction in federal investments. However, personal reasons for exits, which likely intersect with professional ones, may contribute to trends.

## Conclusion

Utilizing longitudinal data from the Current Population Survey, we observe marked increases in exits from the government healthcare workforce and leaving the workforce altogether at both the start of the COVID-19 pandemic and the first half of 2025, corresponding to the start of the Trump Administration. These trends were observed across all levels of government and were most prominent among federal workers. We found similar trends among government behavioral health workers. If these trends continue, the reduction in government healthcare and behavioral health workforces will have significant implications for service delivery and public health capacity that will touch every US community.

## Supplementary Material

qxag032_Supplementary_Data
