# Supplementary material for: Recent trends in US government healthcare & behavioral health workforce departures
Source: Health Aff Sch. 2026 Feb 7;4(2):qxag032. doi: 10.1093/haschl/qxag032 (PMC12931559; doi:10.1093/haschl/qxag032)
Supplement: qxag032_Supplementary_Data [file qxag032_supplementary_data.zip › Appendix.docx]

Appendix Table 1. US Census occupational codes included in study sample

| **Behavioral Health Occupations** | |
| --- | --- |
| 420 | Social and community service managers |
| 1820 | Psychologists |
| 1821 | Clinical and counseling psychologists |
| 1822 | School psychologists |
| 1825 | Other psychologists |
| 2000 | Counselors |
| 2001 | Substance abuse and behavioral disorder |
| 2003 | Marriage and family therapists |
| 2004 | Mental health counselors |
| 2005 | Rehabilitation counselors |
| 2006 | Counselors, all other |
| 2010 | Social workers |
| 2011 | Child, family, and school social worker |
| 2012 | Healthcare social workers |
| 2013 | Mental health and substance abuse social workers |
| 2014 | Social workers, all other |
| 2016 | Social and human service assistants |
| 2025 | Miscellaneous community and social service workers |
| **Healthcare Occupations** | |
| 350 | Medical and health services managers |
| 3000 | Chiropractors |
| 3010 | Dentists |
| 3030 | Dietitians and nutritionists |
| 3040 | Optometrists |
| 3050 | Pharmacists |
| 3060 | Physicians and surgeons |
| 3090 | Other physicians |
| 3100 | Surgeons |
| 3110 | Physician assistants |
| 3120 | Podiatrists |
| 3140 | Audiologists |
| 3150 | Occupational therapists |
| 3160 | Physical therapists |
| 3200 | Radiation therapists |
| 3210 | Recreational therapists |
| 3220 | Respiratory therapists |
| 3230 | Speech-language pathologists |
| 3245 | Therapists, all other |
| 3250 | Veterinarians |
| 3255 | Registered nurses |
| 3256 | Nurse anesthetists |
| 3258 | Nurse practitioners |
| 3260 | Health diagnosing and treating practitioners |
| 3261 | Acupuncturists |
| 3300 | Clinical laboratory technologists and technicians |
| 3310 | Dental hygienists |
| 3320 | Diagnostic related technologists and technicians |
| 3321 | Cardiovascular technologists and technicians |
| 3322 | Diagnostic medical sonographers |
| 3323 | Radiologic technologists and technician |
| 3324 | Magnetic resonance imaging technologist |
| 3330 | Nuclear medicine technologists and medical dosimetrists |
| 3400 | Emergency medical technicians and paramedics |
| 3401 | Emergency medical technicians |
| 3402 | Paramedics |
| 3420 | Health practitioner support technologists |
| 3421 | Pharmacy technicians |
| 3422 | Psychiatric technicians |
| 3423 | Surgical technologists |
| 3430 | Dietetic technicians and ophthalmic med |
| 3500 | Licensed practical and licensed vocational nurses |
| 3510 | Medical records and health information |
| 3515 | Medical records specialists |
| 3520 | Opticians, dispensing |
| 3535 | Miscellaneous health technologists and technicians |
| 3540 | Other healthcare practitioners and technicians |
| 3545 | Miscellaneous health technologists and technicians |
| 3550 | Other healthcare practitioners and technicians |
| 3600 | Nursing, psychiatric, and home health aides |
| 3601 | Home health aides |
| 3602 | Personal care aides |
| 3603 | Nursing assistants |
| 3605 | Orderlies and psychiatric aides |
| 3610 | Occupational therapy assistants and aid |
| 3620 | Physical therapist assistants and aides |
| 3640 | Dental assistants |
| 3645 | Medical assistants |
| 3646 | Medical transcriptionists |
| 3647 | Pharmacy aides |
| 3649 | Phlebotomists |
| 3655 | Healthcare support workers, all other |
| 5730 | Medical secretaries and administrative assistants |
| 8760 | Medical, dental, and ophthalmic laboratories |

Appendix Table 2. Predicted probability of a transition away from government employment, ordered logistic regression (n=66,187)

|  | **Outcome 1** | |  | **Outcome 2** | |  | **Outcome 3** | |
| --- | --- | --- | --- | --- | --- | --- | --- | --- |
|  | No  Transition | |  | Out of Government Transition | |  | Exited the Workforce | |
|  | Pred. Prob. | 95% CI |  | Pred. Prob. | 95% CI |  | Pred. Prob. | 95% CI |
| Year (Oct-Dec/Jan-Jul) |  |  |  |  |  |  |  |  |
| 2015/2016 | 89.2 | [88.3, 90.0] |  | 7.5 | [6.9, 8.1] |  | 3.4 | [3.1, 3.7] |
| 2016/2017 | 89.2 | [88.3, 90.0] |  | 7.5 | [6.9, 8.0] |  | 3.4 | [3.1, 3.7] |
| 2017/2018 | 88.5 | [87.7, 89.4] |  | 7.9 | [7.3, 8.5] |  | 3.6 | [3.3, 3.9] |
| 2018/2019 | 89.1 | [88.2, 90.0] |  | 7.5 | [6.9, 8.1] |  | 3.4 | [3.1, 3.7] |
| 2019/2020 | 88.6 | [87.7, 89.5] |  | 7.8 | [7.2, 8.5] |  | 3.6 | [3.2, 3.9] |
| 2020/2021 | 88.6 | [87.7, 89.5] |  | 7.8 | [7.2, 8.4] |  | 3.6 | [3.2, 3.9] |
| 2021/2022 | 87.9 | [86.9, 88.8] |  | 8.3 | [7.7, 8.9] |  | 3.8 | [3.5, 4.2] |
| 2022/2023 | 89.1 | [88.2, 90.0] |  | 7.5 | [6.9, 8.1] |  | 3.4 | [3.1, 3.7] |
| 2023/2024 | 88.8 | [87.9, 89.7] |  | 7.7 | [7.1, 8.3] |  | 3.5 | [3.2, 3.8] |
| 2024/2025 | 87.9 | [87.0, 88.9] |  | 8.3 | [7.6, 8.9] |  | 3.8 | [3.5, 4.2] |
| Occupation (lagged) |  |  |  |  |  |  |  |  |
| Healthcare Government Worker | 87.7 | [87.3, 88.1] |  | 8.4 | [8.1, 8.7] |  | 3.9 | [3.7, 4.1] |
| Behavioral Health Government Worker | 90.9 | [90.4, 91.4] |  | 6.3 | [6.0, 6.7] |  | 2.8 | [2.6, 3.0] |
| Employer type (lagged) |  |  |  |  |  |  |  |  |
| Local | 88.0 | [87.5, 88.5] |  | 8.2 | [7.9, 8.6] |  | 3.8 | [3.6, 4.0] |
| State | 88.6 | [88.2, 89.0] |  | 7.8 | [7.5, 8.2] |  | 3.6 | [3.4, 3.8] |
| Federal | 90.3 | [89.6, 91.0] |  | 6.7 | [6.3, 7.2] |  | 3.0 | [2.7, 3.3] |
| Race/Ethnicity |  |  |  |  |  |  |  |  |
| White | 89.4 | [89.0, 89.8] |  | 7.3 | [7.0, 7.6] |  | 3.3 | [3.1, 3.5] |
| Black | 87.4 | [86.7, 88.2] |  | 8.6 | [8.1, 9.1] |  | 4.0 | [3.7, 4.3] |
| Latino | 88.3 | [87.5, 89.2] |  | 8.0 | [7.4, 8.6] |  | 3.7 | [3.3, 4.0] |
| Asian | 86.7 | [85.4, 87.9] |  | 9.1 | [8.2, 9.9] |  | 4.3 | [3.8, 4.7] |
| AI/PI/AN/Other/Multi | 89.6 | [88.1, 91.2] |  | 7.2 | [6.1, 8.2] |  | 3.2 | [2.7, 3.8] |
| Education |  |  |  |  |  |  |  |  |
| No College | 83.2 | [82.3, 84.2] |  | 11.3 | [10.7, 12.0] |  | 5.5 | [5.0, 5.9] |
| Some College | 85.6 | [84.6, 86.6] |  | 9.8 | [9.2, 10.5] |  | 4.6 | [4.2, 5.0] |
| Associate's Degree | 88.4 | [87.7, 89.2] |  | 8.0 | [7.4, 8.5] |  | 3.6 | [3.3, 3.9] |
| Bachelor's Degree | 89.5 | [89.0, 90.0] |  | 7.3 | [6.9, 7.7] |  | 3.2 | [3.0, 3.5] |
| Master's Degree | 92.0 | [91.4, 92.5] |  | 5.6 | [5.3, 6.0] |  | 2.4 | [2.2, 2.6] |
| Professional or Doctoral Degree | 90.6 | [89.7, 91.5] |  | 6.6 | [5.9, 7.2] |  | 2.9 | [2.6, 3.2] |
| Citizenship |  |  |  |  |  |  |  |  |
| Not a U.S. Citizen | 85.9 | [84.2, 87.7] |  | 9.6 | [8.4, 10.7] |  | 4.5 | [3.9, 5.2] |
| U.S. Citizen | 88.8 | [88.5, 89.1] |  | 7.7 | [7.5, 8.0] |  | 3.5 | [3.3, 3.7] |
| Martial Status |  |  |  |  |  |  |  |  |
| Not Married | 88.4 | [88.0, 88.9] |  | 8.0 | [7.6, 8.3] |  | 3.6 | [3.4, 3.9] |
| Married | 88.9 | [88.5, 89.3] |  | 7.6 | [7.3, 8.0] |  | 3.5 | [3.3, 3.7] |
| Children |  |  |  |  |  |  |  |  |
| Does Not Have Children | 88.1 | [87.6, 88.5] |  | 8.2 | [7.9, 8.5] |  | 3.8 | [3.5, 4.0] |
| Has Children | 89.3 | [88.9, 89.7] |  | 7.4 | [7.1, 7.7] |  | 3.3 | [3.2, 3.5] |
| Sex |  |  |  |  |  |  |  |  |
| Male | 89.5 | [88.9, 90.0] |  | 7.3 | [6.9, 7.7] |  | 3.3 | [3.1, 3.5] |
| Female | 88.4 | [88.1, 88.8] |  | 7.9 | [7.7, 8.2] |  | 3.6 | [3.5, 3.8] |
| Residence |  |  |  |  |  |  |  |  |
| Does Not Live in Metropolitan Area | 89.3 | [88.5, 90.0] |  | 7.4 | [6.9, 7.9] |  | 3.4 | [3.1, 3.6] |
| Live in Metropolitan Area | 88.6 | [88.3, 88.9] |  | 7.9 | [7.6, 8.1] |  | 3.6 | [3.4, 3.8] |
| Age |  |  |  |  |  |  |  |  |
| 18-35 | 87.2 | [86.6, 87.8] |  | 8.7 | [8.3, 9.2] |  | 4.1 | [3.8, 4.3] |
| 36-50 | 90.1 | [89.6, 90.6] |  | 6.9 | [6.5, 7.2] |  | 3.1 | [2.9, 3.3] |
| 55+ | 88.5 | [88.1, 89.0] |  | 7.9 | [7.5, 8.2] |  | 3.6 | [3.4, 3.8] |
| **Note:** Values shown are the predictive probabilities and 95% confidence interval. | | | | | | | | |

Appendix Figure 1. Transitions away from government employment, by education.

*Source:* Current Population Survey (IPUMS CPS), 2015-2025

Appendix Figure 2. Transitions out of the labor force, by education

*Source:* Current Population Survey (IPUMS CPS), 2015-2025

Appendix Figure 3. Employment transitions among government and non-government healthcare and behavioral health workers

*Source:* Current Population Survey (IPUMS CPS), 2015-2025

*Note*: An employment transition among government workers indicates a shift from government to non-government employment between observation months. An employment transition among non-government workers indicates a change in industry between observation months.

Appendix Figure 4. Labor market exits among government and non-government healthcare and behavioral health workers

*Source:* Current Population Survey (IPUMS CPS), 2015-2025
